# NanoRTax, a real-time pipeline for taxonomic and diversity analysis of nanopore 16S rRNA amplicon sequencing data

**DOI:** 10.1016/j.csbj.2022.09.024

**Published:** 2022-09-23

**Authors:** Héctor Rodríguez-Pérez, Laura Ciuffreda, Carlos Flores

**Affiliations:** a Research Unit, Hospital Universitario Nuestra Señora de Candelaria, Santa Cruz de Tenerife 38010, Spain; b CIBER de Enfermedades Respiratorias, Instituto de Salud Carlos III, Madrid 28029, Spain; c Genomics Division, Instituto Tecnológico y de Energías Renovables (ITER), 38600 Granadilla, Santa Cruz de Tenerife, Spain; d Facultad de Ciencias de la Salud, Universidad Fernando de Pessoa Canarias, 35450 Las Palmas de Gran Canaria, Spain

**Keywords:** NGS, Next-generation sequencing, ONT, Oxford Nanopore Technologies, ICU, Intensive Care Unit, AUC, Area Under the Curve, ROC, Receiver Operating Characteristic curve, Pipeline, Sequencing, Nanopore, Real-time, Microbiome

## Abstract

**Background:**

The study of microbial communities and their applications have been leveraged by advances in sequencing techniques and bioinformatics tools. The Oxford Nanopore Technologies long-read sequencing by nanopores provides a portable and cost-efficient platform for sequencing assays. While this opens the possibility of sequencing applications outside specialized environments and real-time analysis of data, complementing the existing efficient library preparation protocols with streamlined bioinformatic workflows is required.

**Results:**

Here we present NanoRTax, a Nextflow pipeline for nanopore 16S rRNA gene amplicon data that features state-of-the-art taxonomic classification tools and real-time capability. The pipeline is paired with a web-based visual interface to enable user-friendly inspections of the experiment in progress. NanoRTax workflow and a simulated real-time analysis were used to validate the prediction of adult Intensive Care Unit patient mortality based on full-length 16S rRNA sequencing data from respiratory microbiome samples.

**Conclusions:**

This constitutes a proof-of-concept simulation study of how real-time bioinformatic workflows could be used to shorten the turnaround times in critical care settings and provides an instrument for future research on early-response strategies for sepsis.

## Graphical abstract

**Figure:**
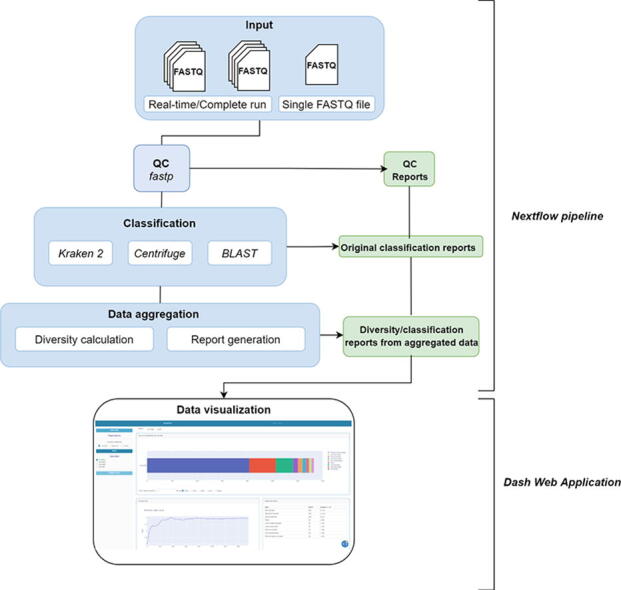

## 1 Introduction

Since the adoption of next-generation sequencing (NGS) technologies, the continuous development of sequencing techniques and cost reductions have revolutionized the study of microbial communities [1]. The ever-growing availability of sequencing equipment in research laboratories and facilities has dramatically increased the number of metagenomic studies, databases, and bioinformatic tools [2], [3]. Consequently, a wide range of applications has emerged in life and health sciences, such as the integration of sequencing approaches in clinical settings [4], [5], where these methods can bolster the speed and sensitivity of traditional microbial culturing and antibiotic susceptibility testing [6].

The introduction of third-generation sequencing technologies, such as Oxford Nanopore Technologies (ONT), has enabled the sequencing of long reads (>1 kbp) while providing a portable platform, which confers the ability to sequence samples even in a non-specialized environment [7], [8]. In particular, ONT long reads can span complete transcripts or genes, and target sequences such as the full-length 16S rRNA gene for taxonomic classification of bacteria. Specifically, the increase in read length has led to a boost in taxonomic resolution and classification accuracy, making it possible to assign reads beyond the genus level when performing pathogen identification or diversity analyses [9]. Besides this, ONT sequencing platforms also feature the unique possibility to access read data of an ongoing experiment in real-time when paired with modern GPU basecalling modes. This characteristic along with the availability of rapid library preparation protocols has served to operate with turnaround times of less than 6 h, a dramatic decrease from the 48–72 h required for microbial culture approaches - emphasizing the potential of bringing a streamlined sequencing and real-time analysis to critical time response settings [10], [11].

This challenge requires pairing rapid laboratory protocols with bioinformatic tools adapted for real-time workflows. Besides, taxonomic classifiers for long reads need to comprehensively evaluate the effect of tool and database selection in a real-time analysis scenario [12]. Here we present NanoRTax, a nextflow-based pipeline for bacterial taxonomy classification and sample diversity analysis of nanopore full-length 16S rRNA amplicon reads. The pipeline features the integration of state-of-the-art read classification methods, downstream analysis, and real-time capability to enable benchmarking of 16S rRNA gene classification methods while the sequencing experiment is in progress. The pipeline is paired with an independent Dash web application which provides immediate access to taxonomic information, diversity statistics, and visualizations.

## 2 Materials and methods

NanoRTax is implemented in the Nextflow [13] workflow management system to enable efficient parallel execution and built-in integration of software dependencies using Docker containers and conda environments.

NanoRTax input consists of basecalled and demultiplexed FASTQ files following the structure of MinKNOW sequencing software output directories. The output path of an ongoing experiment can be specified for real-time analysis of newly generated FASTQ files. First, input sequences undergo a quality control step using Fastp [14]. By default, reads of length below 1400 base pairs (bp) or above 1700 bp are discarded to keep only near full-length 16S rRNA sequences. However, these can be specified manually by the user for alternative length intervals. Then, taxonomic assignment is performed by one or more classifiers of choice between Kraken2 [15], Centrifuge [16], and BLAST [17]. Database and parameter selection for each tool can be specified via command line or in pre-loaded configuration files, and users can easily change the database for another of their choice. The classification output is then processed to extract the full taxonomy for every classified read using Taxonkit [18]. This information is used in the next step to generate the NanoRTax final output that includes the sequence relative abundances, diversity index calculations at different taxonomic levels and an abundance table with taxons for each sample analyzed on the execution. A report aggregation step is performed while new FASTQ sequence files are fed to the pipeline and further classified. This enables the synchronization of NanoRTax execution with the sequencing experiment and allows the inspection of partial results of the ongoing experiment.

**Fig. 1:**
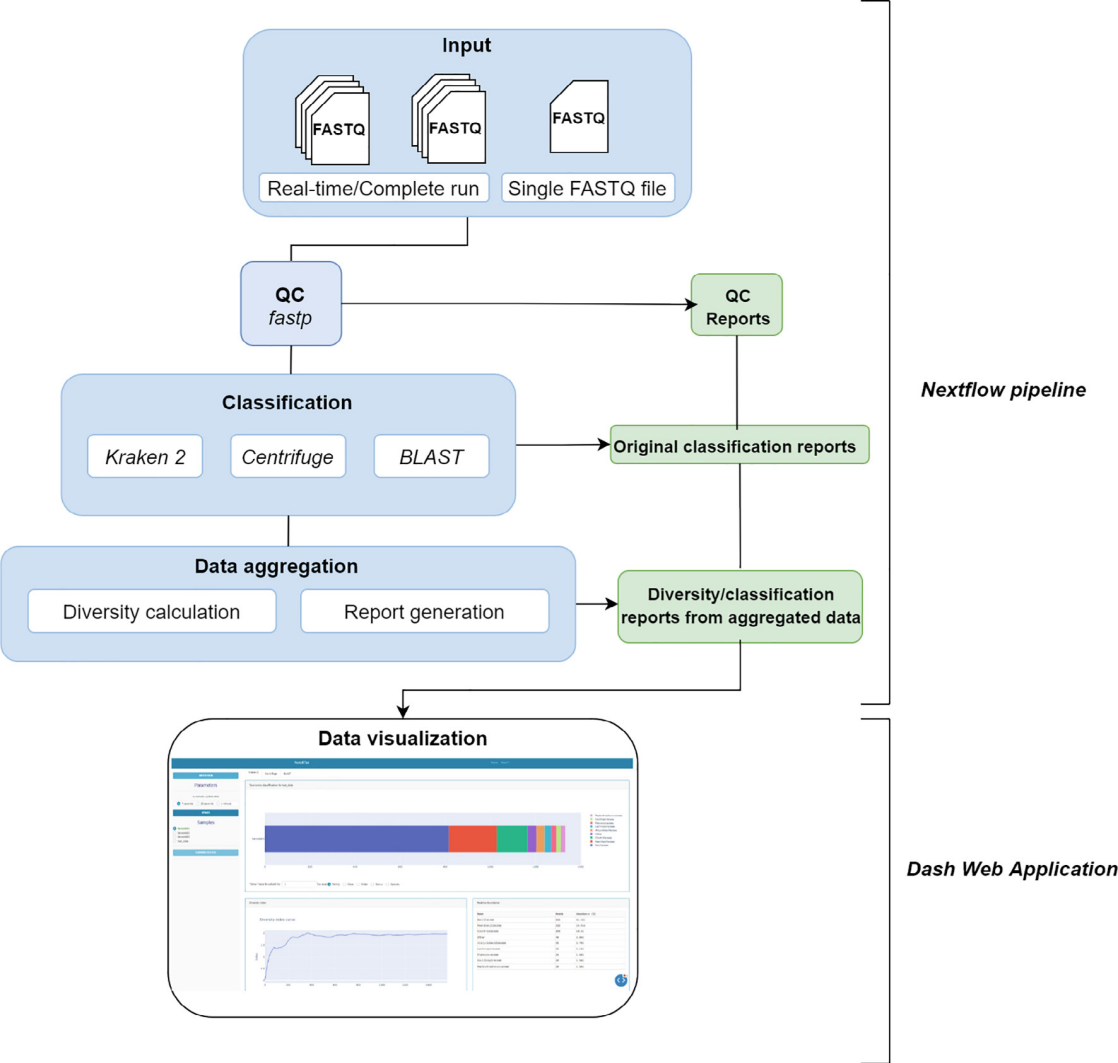
NanoRTax general overview.

For user-friendly visualization of the partial or complete outputs, the pipeline can be paired with an independent Python Dash web application, which serves as a dashboard to explore outputs in real time. The interface integrates interactive summary tables and plots regarding quality control parameters, relative abundances with modifiable frequency cutoffs, and sample diversity index calculations over time. The general workflow of NanoRTax and software versions are detailed in Fig. 1 and Table 1.

**Table 1:** NanoRTax software dependencies and versions.

Fastp	v0.20.1
Kraken2	v1.1.1
Centrifuge	v1.0.4 beta
blastn	v2.11.0
Taxonkit	v0.8.0

## 3 Results

To assess the usefulness of NanoRTax real-time analysis capability, we analyzed the full-length 16S rRNA gene nanopore sequencing data from 31 tracheal aspirates from adult Intensive Care Unit (ICU) patients with non-pulmonary sepsis (n = 31, 25 survivors and six deceased patients) collected from a single medical-surgical ICU at sepsis diagnosis (within 8 h). We previously showed that this small cohort had sufficient statistical power and described that a reduction in genus-level bacterial lung diversity within 8 h of sepsis diagnosis is associated with ICU mortality, providing a potentially novel and early prognostic biomarker of non-pulmonary sepsis with better prognostic ability than other commonly used clinical scores [19]. We performed the re-analysis of this data using the NanoRTax complete workflow and generated the patient-level reports [20].

**Fig. 2:**
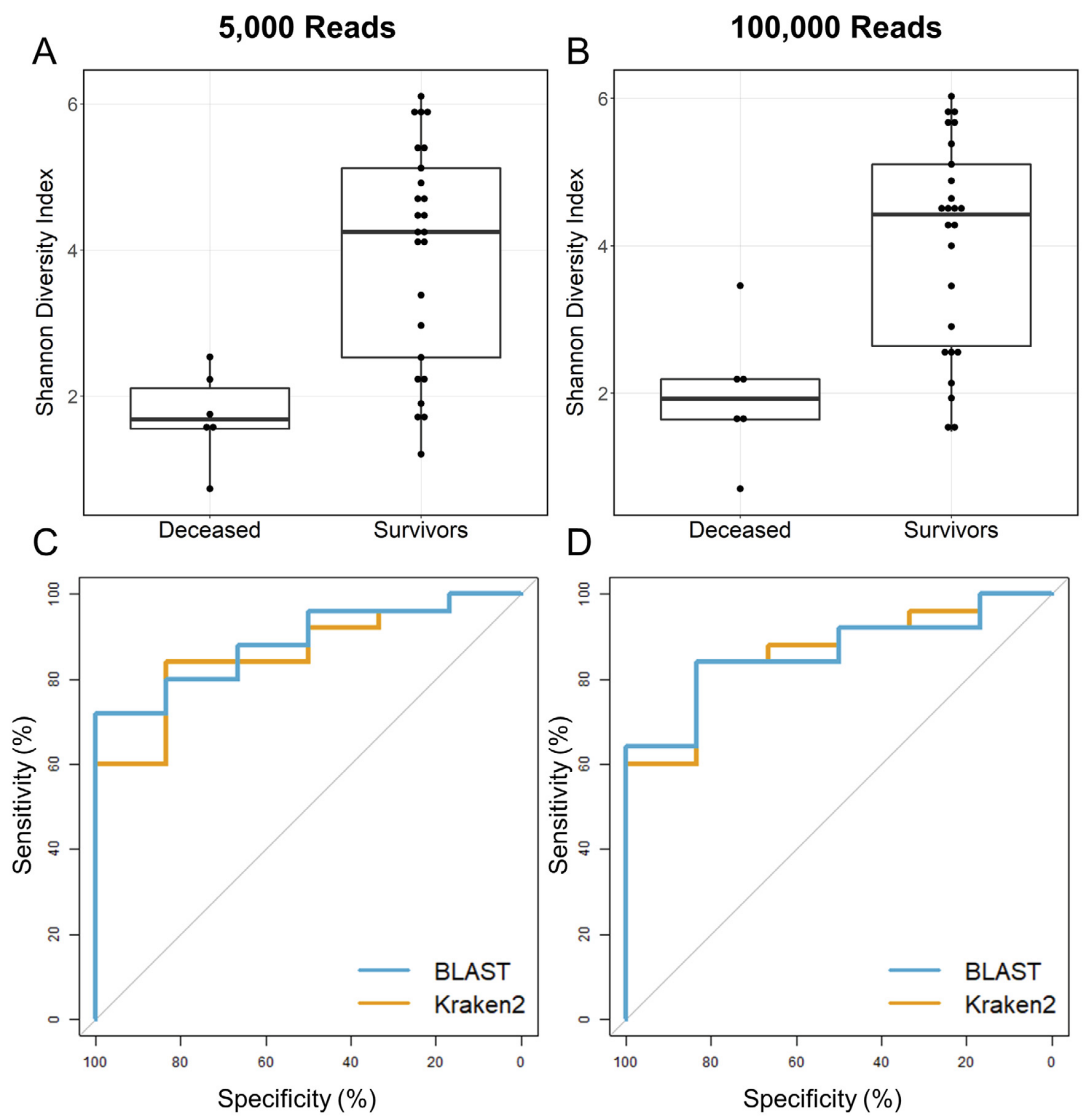
Boxplots of the Shannon diversity index per patient sample calculated with 5,000 reads (**A**) and 100,000 reads (**B**). Receiver Operating Characteristic (ROC) curves of mortality of ICU patients calculated with 5,000 reads (**C**) and 100,000 reads (**D**) for Kraken2 (orange) and BLAST (blue) classifiers at species level based on 8 h lung dysbiosis. (For interpretation of the references to colour in this figure legend, the reader is referred to the web version of this article.)

For each ICU sample, species-level diversity index metrics were calculated periodically from 5,000 to 100,000 reads to simulate different time periods of an ongoing sequencing experiment. Shannon diversity index calculated at species level for each time period was then compared between deceased and survivor patients based on Kraken2 and the NCBI RefSeq database containing only bacterial genomes. BLAST classifications based on NCBI 16S RefSeq database were used only for reference as this combination of classifier and database provided the best performance in our previous assessments [21]. The predictive value of the lung bacterial diversity index was assessed by fitting a linear model and calculating the Area Under the Curve (AUC) from Receiver Operating Characteristic (ROC) curves (Fig. 2). We observed a reduction in the Shannon diversity index in deceased ICU patients compared to survivors as early as at less than 2 h of the simulated sequencing experiment, which roughly corresponds to 5,000 reads (Wilcoxon test, *p* = 0.002 and AUC = 88.67 % (Kraken2); 86.00 % (BLAST)).

These results were essentially equivalent to those obtained in a simulated experiment collecting reads for 24–48 h, roughly corresponding to 100,000 reads (Wilcoxon test, *p* = 0.005 and AUC = 88.67 % (Kraken2); 86.00 % (BLAST)).

## 4 Discussion

The strong association of reduced lung bacterial diversity with a worse sepsis prognosis highlights the importance of host-microbial interactions and provides an early prognostic biomarker for sepsis. An early sepsis response has been proven to be of paramount importance for patient outcomes improvement and will remain relevant until novel drugs or interventions are demonstrated to be effective [22]. Applications in the context of diagnosis and mortality prediction have been explored recently, aiming to integrate not only sequencing information but also clinical data to enable better diagnosis, prognosis prediction, or entailment of treatment [23], [24], [25]. In this study, we simulated a realistic scenario of a real-time framework to predict ICU mortality in sepsis patients based on 16S rRNA gene sequencing experiments on lung samples paired with rapid analysis protocols, allowing us to draw the same conclusions as those from a complete 48 h sequencing dataset. Moreover, these results validated the previously observed lung dysbiosis association with mortality [19] to the species level as a result of the higher taxonomic resolution achieved by sequencing the full-length 16S rRNA genes. NanoRTax enables the immediate analysis of data while sequencing by implementing Kraken2 and Centrifuge rapid classifiers, which have been recently evaluated for long-read metagenomic profiling [26], [27]. Additionally, the taxonomic assignment can be performed with BLAST to provide a framework to benchmark the tool in a real-time context or to evaluate Kraken2 and Centrifuge tools against a gold-standard BLAST classification. Our results also serve as a proof-of-concept of how real-time bioinformatic workflows could be useful to shorten the turnaround times in critical care settings and suggest their possible use for future research on early-response strategies for sepsis.

While NanoRTax was designed for full-length 16S rRNA gene taxonomic analysis of microbial samples, a focus on different amplification targets and the use of pipeline parametrization could take the application beyond bacterial profiling. Similar NanoRTax-based classification workflows can be proposed for the detection of fungal and viral infections [28], [29], while non-taxonomic targeted amplicons can profile either specific antimicrobial resistance genes or entire gene panels such as the resistome [30]. Furthermore, continuous releases of the ONT sequencing chemistry and improvements in the basecalling algorithms are expected to positively impact taxonomic assignments using NanoRTax. ONT hardware releases like the ONT Flongle sequencing adapter or the ONT Voltrax library preparation device can simplify rapid portable sequencing workflows by reducing the resources needed for the experimental protocols [31]. Enhanced portability and analytical speed directly benefit the in-situ assessment of microbial samples and confer relevance to the real-time bioinformatics tools described in this study. However, there are substantial practical challenges for routine taxonomic classification and metagenomics applications outside research practices [32]. Both analytical factors, such as sensitivity limitations due to genome size, pathogen load, or ease of microorganism lysis [33], and sample factors, such as background contamination issues [34], [35] can affect classification results in metagenomics studies. Bioinformatic analysis also turned out to be non-trivial since the completeness and accuracy of the ever-growing sequence databases and different approaches of taxonomic methods have been demonstrated to have an important effect on results [2], [36], [37]. Thus, careful interpretation and constant benchmarking of analysis methods and databases will be key for taxonomic classification and metagenomic application success [38]. In order to successfully take data analysis to a real-time scenario, GPU basecalling of raw data generated from an ONT sequencing experiment is necessary to enable streaming bioinformatic analysis. In fact, both fast and high accuracy basecalling models on CPU mode are too slow when approaching real-time applications (e.g., basecalling of 4,000 16S rRNA gene reads using fast mode took 7–13 min using 48 CPU threads *vs* 10–15 s using GPU in our settings).

## 5 Conclusions

We have developed NanoRTax, a bioinformatics pipeline to enable real-time taxonomic analysis of full-length 16S rRNA nanopore reads featuring multiple classification tools and immediate output visualization. We applied the NanoRTax workflow to the evaluation of 16S rRNA gene sequencing data of lung samples aimed to predict mortality in sepsis patients admitted to the ICU. Despite further experimental demonstrations are needed, our results obtained from the analysis of simulated very early sequencing data (within 2 h) support the benefits of implementing NGS-based assessments in this scenario. Despite this field is experiencing a fast development pace, we expect that routine clinical metagenomics will remain outside critical time-response scenarios until limitations are addressed. We anticipate that real-time bioinformatic analysis tools and implementations will be advancing concurrently with NGS development and applications.

## 6 Availability and requirements

*Project name*: NanoRTax.

*Project home page*: https://github.com/genomicsITER/NanoRTax.

*Operating systems*: Linux, Mac.

*Programming language*: Nextflow, Python.

*Other requirements*: Java 8 or higher, Conda 4.10.or higher or Docker 1.6.2.

*License*: MIT.

*Any restrictions to use by non-academics*: License needed.

## 7 Declarations

### 7.1 Ethics approval and consent to participate

Not applicable.

### 7.2 Consent for publication

Not applicable.

### 7.3 Availability of data and materials

NanoRTax Nextflow pipeline and Python Dash web application are freely available under MIT license on Github [39] (https://github.com/genomicsITER/NanoRTax). The repository includes instructions and a testing dataset for a minimal pipeline execution.

## Funding

This work was supported by Instituto de Salud Carlos III [PI14/00844, PI17/00610, and FI18/00230] and co-financed by the European Regional Development Funds, “A way of making Europe” from the European Union; Ministerio de Ciencia e Innovación [RTC-2017–6471-1, AEI/FEDER, UE]; Cabildo Insular de Tenerife [CGIEU0000219140]; Fundación Canaria Instituto de Investigación Sanitaria de Canarias [PIFUN48/18]; and by the agreement with Instituto Tecnológico y de Energías Renovables (ITER) to strengthen scientific and technological education, training, research, development and innovation in Genomics, Personalized Medicine and Biotechnology [OA17/008].

## Author contributions

HRP coded NanoRTax pipeline and web application, performed bioinformatic analysis and drafted the manuscript. LC performed the microbiome samples library preparation, sequencing and was a major contributor in testing, data analysis, and writing the manuscript. CF supervised the project concept and study design, contributed to data analysis and all drafts, and obtained funding. All authors read and approved the final manuscript.

## Declaration of Competing Interest

The authors declare that they have no known competing financial interests or personal relationships that could have appeared to influence the work reported in this paper.
